# Evaluation of Cardiovascular Responses to Endotracheal Intubation With Alkalinized Lignocaine and Air Cuff Inflation Techniques

**DOI:** 10.7759/cureus.89734

**Published:** 2025-08-10

**Authors:** Rajanikanth Kanumetta, Padma Amar Vishal, Rudraraju Sri Soumya, Thoyaja Devi Vangapandu, Dathrika Vagdevi Kotturu, Suresh Babu Sayana

**Affiliations:** 1 Anaesthesiology, Central hospital, Coal India Limited, Brajarajnagar, IND; 2 Anaesthesiology, NRI Institute of Medical Sciences, Visakhapatnam, IND; 3 Anaesthesiology, Konaseema Institute of Medical Sciences and Research Foundation, Amalapuram, IND; 4 Anaesthesiology, GSL Medical College, Rajahmundry, IND; 5 Anaesthesiology, Area Hospital, Vizianagaram, IND; 6 Department of Pharmacology, Government Medical College and General Hospital, Palvancha, IND

**Keywords:** alkalinized lignocaine, cuff pressure control, endotracheal intubation, general anesthesia, hemodynamic response

## Abstract

Background

Endotracheal intubation is a routine but critical aspect of airway management during general anesthesia. However, it frequently triggers reflex sympathetic stimulation due to mechanical irritation of the laryngotracheal structures, leading to notable increases in heart rate and blood pressure. These hemodynamic changes can be particularly concerning in patients with underlying cardiovascular disorders such as hypertension or ischemic heart disease. This study was designed to assess the efficacy of alkalinized lignocaine for endotracheal cuff inflation in attenuating these responses, compared to conventional air inflation.

Methods

This prospective, randomized, comparative clinical study enrolled 60 adult patients scheduled for elective surgical procedures under general anesthesia. Participants were randomly assigned to two equal groups. In Group A, the endotracheal tube cuff was inflated with air, while in Group B, the cuff was filled with 2% lignocaine alkalinized using 8.4% sodium bicarbonate in a standardized 19:1 ratio. In both groups, cuff inflation was performed until no audible air leak was detected on auscultation, and the cuff pressure was subsequently adjusted to approximately 25 cmH₂O using a cuff pressure manometer to ensure consistency and minimize mucosal trauma. Hemodynamic parameters - including heart rate (HR), systolic blood pressure (SBP), diastolic blood pressure (DBP), and mean arterial pressure (MAP) - were meticulously recorded at baseline and at 1, 3, 5, and 10 minutes following tracheal intubation. Data were analyzed using appropriate statistical tests, with a p-value < 0.05 considered statistically significant.

Results

Baseline demographic and clinical characteristics were comparable between the two groups, with no statistically significant differences prior to intervention. Group B demonstrated significantly attenuated increases in HR, SBP, DBP, and MAP at all post-intubation time points, with the most pronounced reduction observed at the 1-minute mark. For instance, HR at 1-minute post-intubation averaged 103.4 ± 8.6 bpm in Group A versus 91.2 ± 7.3 bpm in Group B (p < 0.001). Additionally, patients in Group B achieved near-baseline hemodynamic values more rapidly than those in Group A.

Conclusion

Inflation of the endotracheal tube cuff with alkalinized lignocaine is a simple, effective, and noninvasive technique to attenuate the hemodynamic stress response associated with endotracheal intubation. Incorporating this method into routine anesthetic practice may improve cardiovascular stability, particularly in high-risk patients.

## Introduction

Endotracheal intubation remains a cornerstone in airway management during general anesthesia and critical care procedures [1, 2]. However, the mechanical stimulation caused by laryngoscopy and tracheal tube insertion is known to trigger a marked sympathetic surge, resulting in transient but significant elevations in heart rate (HR) and blood pressure (BP) [3, 4]. Although transient, these cardiovascular responses may precipitate clinically significant complications in individuals with pre-existing conditions such as ischemic heart disease, systemic hypertension, or cerebrovascular disorders.

These hemodynamic responses are principally attributed to the stimulation of mechanoreceptors within the laryngotracheal region, compounded by the compressive force exerted by the inflated endotracheal tube cuff against the tracheal mucosa [5]. Multiple approaches have been proposed to mitigate these effects, ranging from pharmacologic agents such as opioids and beta-blockers to airway interventions like supraglottic devices [6]. Among these, the use of lignocaine has gained popularity due to its dual role as a local anesthetic and anti-arrhythmic agent [7, 8].

When the endotracheal cuff is inflated with lignocaine, the drug can diffuse through the cuff’s semi-permeable wall and act directly on the tracheal lining [9]. This provides localized anesthesia, which helps suppress afferent nerve transmission and diminishes the reflex sympathetic output [8-10]. The clinical efficacy of this method can be enhanced by alkalinizing lignocaine with sodium bicarbonate, which enhances the non-ionized fraction of the drug, allowing for faster and more efficient mucosal penetration [10].

Previous studies have highlighted the benefits of intracuff lignocaine in minimizing postoperative discomfort, such as coughing and sore throat [11, 12]. However, there is limited evidence evaluating its role in modulating acute hemodynamic fluctuations during intubation. This study was therefore designed to compare the hemodynamic outcomes, specifically HR, systolic blood pressure (SBP), diastolic blood pressure (DBP), and mean arterial pressure (MAP), in patients undergoing endotracheal intubation with cuffs inflated using either air or alkalinized lignocaine.

The principal aim of this investigation was to compare the immediate post-intubation hemodynamic responses - including HR, SBP, DBP, and mean arterial pressure - between adult patients undergoing elective surgery under general anesthesia, in whom endotracheal tube cuffs were inflated with either air or alkalinized lignocaine.

## Materials and methods

Study design and ethical considerations

This randomized, prospective, comparative study was conducted at the Konaseema Institute of Medical Sciences (KIMS), Amalapuram, between January 2021 and September 2022. The study commenced after receiving approval from the Institutional Ethics Committee under the ethical approval number Serial No. of IEC/PR/2021 118/02.12.2020 dated December 3, 2020. As the study was observational and not categorized as a clinical trial under the Indian regulatory framework, it was not registered with the Clinical Trial Registry of India (CTRI). Written informed consent was obtained from all participants before their inclusion in the study, in accordance with ethical principles outlined in the Declaration of Helsinki.

Sample size calculation

Determination of Sample Size

The sample size for the study was determined in advance to ensure that the statistical analysis would be sufficiently powered to detect clinically relevant differences in hemodynamic parameters between the two study groups. The primary variables of interest were HR and MAP, which are commonly used indicators of cardiovascular response to endotracheal intubation. Drawing from previously published literature that demonstrated hemodynamic variability based on different cuff inflation media, the minimum number of participants required in each group was estimated. To enhance the reliability of the study outcomes and account for the possibility of patient attrition or protocol deviations, the sample size was increased beyond the calculated minimum.

Power Analysis

A formal power analysis was conducted using G*Power software version 3.1.9.7 (Heinrich Heine University, Düsseldorf, Germany). The calculation was based on an anticipated effect size (Cohen’s d) of 0.8, which reflects a medium to large effect size as per conventional statistical thresholds. To achieve a statistical power of 80% (β = 0.20) with a two-tailed alpha error of 0.05, the minimum required sample size was estimated to be 26 subjects in each group for a two-group comparison of means using an independent samples t-test. To strengthen the study’s statistical validity and accommodate any unforeseen exclusions, the sample size was rounded up to 30 participants per group, yielding a total sample of 60 patients included in the study.

Participants

A total of 60 adult patients, aged 18 to 60 years, classified as American Society of Anesthesiologists (ASA) physical status I or II, were enrolled in this study. All patients were scheduled for elective surgical procedures under general anesthesia requiring endotracheal intubation.

Patient Selection

Patients were excluded if they had a predicted difficult airway (Modified Mallampati Class III or IV), known hypersensitivity to lignocaine or sodium bicarbonate, active upper or lower respiratory tract infections, chronic obstructive pulmonary disease, or significant cardiovascular instability such as arrhythmias or uncontrolled hypertension. Additional exclusion criteria included the use of medications known to influence hemodynamic responses (e.g., beta-blockers), established cardiovascular comorbidities including hypertension and ischemic heart disease, and anticipated need for prolonged intubation exceeding two hours. These contraindications were carefully defined to minimize confounding variables, enhance patient safety, and ensure consistency in evaluating the hemodynamic effects of endotracheal cuff inflation media.

Randomization and group allocation

**Figure 1:**
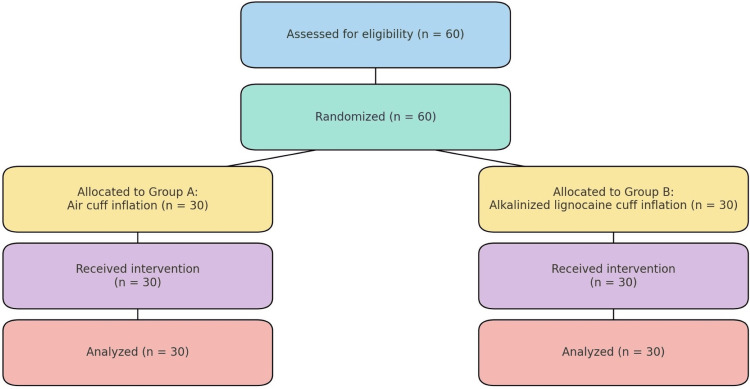
CONSORT Flow Diagram of Patient Enrollment, Allocation, Intervention, and Analysis

Patients were randomly allocated into two groups using a computer-generated randomization sequence to ensure unbiased distribution. Group assignments were concealed using the sequentially numbered, opaque, sealed envelopes (SNOSE) method to maintain allocation concealment. Group A (Air Group) consisted of 30 patients whose endotracheal tube (ETT) cuffs were inflated with air. Group B (Lignocaine Group) included 30 patients in whom the ETT cuffs were inflated with a freshly prepared solution of 2% lignocaine hydrochloride (Lox 2%), alkalinized with 8.4% sodium bicarbonate injection in a standardized 19:1 volume ratio, prepared immediately before use. The average volume of solution used ranged between 2 and 4 mL, corresponding to a total lignocaine dose of 40-80 mg, which is well within the recommended maximum dose for mucosal application (3-4 mg/kg). This ensured both safety and effective diffusion across the cuff to provide tracheal mucosal anesthesia. The detailed participant flow is illustrated in the CONSORT diagram (Figure 1).

Anesthetic protocol

All participants received a standardized premedication regimen consisting of intravenous midazolam at 0.05 mg/kg and glycopyrrolate at 0.004 mg/kg. Continuous intraoperative monitoring was instituted using electrocardiography (ECG), non-invasive blood pressure (NIBP), and peripheral oxygen saturation (SpO₂), in accordance with ASA monitoring standards. General anesthesia was induced with intravenous propofol at 2 mg/kg and fentanyl at 2 µg/kg to achieve adequate hypnosis and analgesia. Neuromuscular blockade was facilitated with intravenous vecuronium at 0.1 mg/kg. Tracheal intubation was performed three minutes following induction, using a standard Macintosh laryngoscope with the patient in the sniffing position. An appropriately sized, high-volume, low-pressure cuffed endotracheal tube was used for all patients to minimize mucosal trauma and ensure a consistent and effective airway seal. To maintain procedural uniformity and reduce inter-operator variability, all intubations were performed by a single experienced anesthesiologist with a minimum of three years of postgraduate clinical experience.

Preoperative airway assessment was conducted using the Modified Mallampati classification, and only patients with Class I or II scores were included to exclude anticipated difficult airways. In Group A, the endotracheal cuff was inflated with air. In Group B, the cuff was inflated with freshly prepared alkalinized lignocaine, obtained by mixing 2% lignocaine hydrochloride with 8.4% sodium bicarbonate in a standardized 19:1 volume ratio immediately before administration. The mixture was instilled into the cuff using a sterile syringe until no audible air leak was detected on auscultation at the suprasternal notch. In both groups, the final cuff pressure was measured using a handheld aneroid cuff pressure manometer and adjusted to a standardized target of 25 cmH₂O to ensure effective sealing while minimizing the risk of tracheal mucosal ischemia or pressure-related complications.

Biomechanically, endotracheal cuffs made from polyvinyl chloride (PVC) are semi-permeable and allow passive diffusion of non-ionized lignocaine into the tracheal mucosa when alkalinized. This local diffusion produces topical anesthesia, effectively reducing mucosal irritation and reflex sympathetic activation during intubation. Available literature indicates that such administration does not compromise cuff integrity, lead to structural degradation, or promote biofilm formation during short-term intraoperative use (up to 2-3 hours). No adverse cuff-related mechanical complications (e.g., leakage or rupture) were observed.

Hemodynamic monitoring

Hemodynamic parameters - including HR, SBP, DBP, and MAP - were meticulously recorded at baseline (prior to induction), immediately after intubation, and subsequently at 1, 3, 5, and 10 minutes following tracheal intubation to assess the physiological response over time.

To ensure standardization and patient safety, the cuff pressure was monitored in all cases using a handheld aneroid cuff pressure manometer. After initial inflation with either air or alkalinized lignocaine solution, the cuff pressure was adjusted to a uniform target of 25 cmH₂O in both groups to achieve an effective seal and reduce the risk of tracheal mucosal injury. Additionally, all hemodynamic parameters-including HR, SBP, DBP, and MAP-were recorded by a trained observer who was blinded to group allocation. This individual was not involved in the intubation procedure or anesthetic administration, thereby minimizing observer bias in outcome measurements.

Statistical analysis

Data were analyzed using SPSS software, version 26.0 (IBM Corp., Armonk, NY, USA). Continuous variables were expressed as mean values with standard deviations (SD), and comparisons between the two groups were performed using independent samples t-tests. Categorical variables were evaluated using the chi-square (χ²) test. Throughout the analysis, a p-value of less than 0.05 was considered indicative of statistical significance.

## Results

Demographic

A total of 60 adult patients scheduled for elective surgical procedures under general anesthesia were enrolled and randomly allocated into two equal groups (n = 30 each). Group A received endotracheal tube cuff inflation with air, while Group B received cuff inflation with alkalinized lignocaine solution. Baseline demographic variables, including age, sex distribution, and ASA physical status classification, were comparable between the two groups. The mean age was 36.8 ± 9.4 years in Group A and 35.5 ± 8.7 years in Group B, with no statistically significant difference (t = 0.56, p = 0.5804). Gender distribution was also similar, with 17 males and 13 females in Group A and 15 males and 15 females in Group B (χ² = 0.07, p = 0.7958). The proportion of ASA Class I and II patients was nearly identical between the groups (16/14 in Group A vs. 17/13 in Group B), showing no significant variation (χ² = 0.00, p = 1.0000). These results confirm the demographic equivalence of the study groups, ensuring comparability for further hemodynamic assessments (Table 1).

**Table 1:** Demographic Characteristics of the Study Population (n = 60) Independent samples t-test for age; Chi-square (χ²) test for gender and ASA status.

Variable	Group A (Air, n = 30)	Group B (Alkalinized Lignocaine, n = 30)	Test Statistic Value (t or χ²)	p-value
Age (years)	36.8 ± 9.4	35.5 ± 8.7	t = 0.56	0.5804
Male/Female	17/13	15/15	χ² = 0.07	0.7958
ASA I / II	16/14	17/13	χ² = 0.00	1.0000

Heart rate (HR) changes

Following tracheal intubation, both groups exhibited an increase in HR relative to their respective baseline values. The baseline HR was similar between groups (80.5 ± 5.1 bpm in Group A vs. 81.2 ± 5.1 bpm in Group B; p = 0.5971), suggesting comparable pre-induction cardiovascular status. At 1-minute post-intubation, Group A experienced a sharp rise in HR, peaking at 103.4 ± 8.0 bpm, whereas Group B recorded a significantly attenuated peak of 91.2 ± 8.0 bpm (t = 5.92, p < 0.0001). This trend persisted over subsequent time intervals, with Group A maintaining higher HR values at 3 minutes (98.2 ± 7.2 bpm) compared to Group B (87.9 ± 7.2 bpm; p < 0.0001), and at 5 minutes (91.7 ± 6.2 bpm vs. 83.6 ± 6.2 bpm; p < 0.0001). By the 10-minute mark, the HR in both groups had nearly returned to baseline, yet Group B continued to show significantly lower values (85.3 ± 4.9 bpm vs. 80.1 ± 4.9 bpm; p = 0.0001). These findings confirm that intracuff alkalinized lignocaine effectively attenuates the tachycardic response associated with laryngoscopy and intubation (Table 2).

**Table 2:** Comparison of HR (beats per minute) Between Groups at Various Time Intervals Statistical test: Independent samples t-test

Time	Group A (Air, n = 30)	Group B (Alkalinized Lignocaine, n = 30)	t-value	p-value
Baseline	80.5 ± 5.1	81.2 ± 5.1	-0.53	0.5971
1 min	103.4 ± 8.0	91.2 ± 8.0	5.92	<0.0001
3 min	98.2 ± 7.2	87.9 ± 7.2	5.57	<0.0001
5 min	91.7 ± 6.2	83.6 ± 6.2	5.10	<0.0001
10 min	85.3 ± 4.9	80.1 ± 4.9	4.07	0.0001

Comparison of SBP (mmHg) between groups

Both groups exhibited comparable baseline SBP values, with Group A recording 122.6 ± 6.3 mmHg and Group B recording 121.9 ± 6.3 mmHg (p = 0.6687), indicating no significant pre-induction difference. However, following endotracheal intubation, Group A showed a marked rise in SBP, reaching a peak of 148.7 ± 9.0 mmHg at 1 minute. In contrast, Group B demonstrated a significantly lower SBP of 132.5 ± 9.0 mmHg at the same time point (t = 7.01, p < 0.0001). This attenuated hypertensive response in the lignocaine group continued at 3 minutes (140.3 ± 8.3 mmHg in Group A vs. 126.8 ± 8.3 mmHg in Group B; p < 0.0001) and 5 minutes (130.8 ± 7.2 mmHg vs. 123.4 ± 7.2 mmHg; p = 0.0002). Although the difference narrowed by 10 minutes post-intubation, SBP remained significantly lower in Group B (125.6 ± 6.0 mmHg in Group A vs. 120.3 ± 6.0 mmHg in Group B; p = 0.0012). These results highlight the superior hemodynamic stability achieved with intracuff alkalinized lignocaine (Table 3).

**Table 3:** Comparison of SBP (mmHg) Between Groups at Various Time Intervals Statistical test: Independent samples t-test

Time	Group A (Air, n = 30)	Group B (Alkalinized Lignocaine, n = 30)	t-value	p-value
Baseline	122.6 ± 6.3	121.9 ± 6.3	0.43	0.6687
1 min	148.7 ± 9.0	132.5 ± 9.0	7.01	<0.0001
3 min	140.3 ± 8.3	126.8 ± 8.3	6.3	<0.0001
5 min	130.8 ± 7.2	123.4 ± 7.2	3.98	0.0002
10 min	125.6 ± 6.0	120.3 ± 6.0	3.42	0.0012

Comparison of DBP (mmHg) between groups

A similar trend was observed in DBP between the two groups. Both groups started with comparable baseline DBP values (78.4 ± 5.3 mmHg in Group A vs. 79.1 ± 5.3 mmHg in Group B; p = 0.6362), indicating no pre-intubation difference. However, following endotracheal intubation, Group A exhibited a more pronounced increase in DBP at 1 minute (92.6 ± 6.3 mmHg), whereas Group B showed a significantly lower value (81.3 ± 6.3 mmHg), with a t-value of 6.16 and p < 0.0001. This attenuated response in Group B persisted throughout the subsequent time points at 3-, 5-, and 10-minute post-intubation, with all comparisons showing statistically significant reductions (p < 0.001). These findings support the efficacy of intracuff alkalinized lignocaine in reducing the diastolic pressor response during laryngoscopy and tracheal intubation (Table 4).

**Table 4:** Comparison of DBP (mmHg) Between Groups at Various Time Intervals Statistical test: Independent samples t-test

Time	Group A (Air, n = 30)	Group B (Alkalinized Lignocaine, n = 30)	t-value	p-value
Baseline	78.4 ± 5.6	79.1 ± 5.6	-0.48	0.6362
1 min	92.6 ± 7.1	81.3 ± 7.1	6.16	<0.0001
3 min	89.2 ± 6.7	78.5 ± 6.7	6.23	<0.0001
5 min	83.5 ± 6.0	76.4 ± 6.0	4.58	<0.0001
10 min	80.1 ± 5.3	75.2 ± 5.3	3.58	0.0007

Comparison of MAP (mmHg) between groups

Table 5 presents the comparison of MAP between Group A (cuff inflated with air) and Group B (cuff inflated with alkalinized lignocaine) at various time intervals following endotracheal intubation. At baseline, MAP values were comparable between the groups (93.1 ± SD vs. 92.6 ± SD; p = 0.7521), indicating no significant pre-intervention difference. However, at 1-minute post-intubation, Group A exhibited a marked rise in MAP (111.3 ± SD), while Group B showed a significantly attenuated response (100.2 ± SD), with a t-value of 5.21 and a p-value < 0.0001. This trend of reduced MAP elevation in Group B persisted at 3-, 5-, and 10-minute post-intubation, with all time points demonstrating statistically significant differences (p < 0.05). These findings suggest that intracuff alkalinized lignocaine effectively blunted the pressor response associated with laryngoscopy and intubation, offering improved hemodynamic stability compared to air inflation.

**Table 5:** Comparison of MAP (mmHg) Between Groups Statistical test: Independent samples t-test

Time	Group A (Air, n = 30)	Group B (Alkalinized Lignocaine, n = 30)	t-value	p-value
Baseline	93.1 ± 6.1	92.6 ± 6.1	0.32	0.7521
1 min	111.3 ± 8.3	100.2 ± 8.3	5.21	<0.0001
3 min	106.2 ± 7.6	96.1 ± 7.6	5.14	<0.0001
5 min	98.3 ± 6.6	92.3 ± 6.6	3.52	0.0008
10 min	94.7 ± 5.7	90.8 ± 5.7	2.63	0.011

Adverse effects

Patients in both groups were observed for a duration of 10 minutes following endotracheal intubation, during which hemodynamic parameters and any clinically significant adverse effects were continuously monitored. Throughout this observation window, no adverse events such as arrhythmias, hypotension, bradycardia, or allergic reactions were recorded in either group. The monitoring was conducted as part of standard intraoperative surveillance using ECG, NIBP, and SpO₂. To reduce potential bias, the adverse effect assessment was performed by an independent observer who was blinded to group allocation. This ensured objectivity in the reporting of untoward events and enhanced the methodological rigor of the study.

## Discussion

The present study confirms that endotracheal cuff inflation with alkalinized lignocaine significantly attenuates the acute hemodynamic responses triggered by laryngoscopy and intubation, as compared to air inflation. Patients in the lignocaine group demonstrated less pronounced tachycardic and pressor responses, with faster return to near-baseline values across all measured time points. These findings indicate that intracuff alkalinized lignocaine offers superior cardiovascular stability during the peri-intubation period, making it a valuable strategy in patients at risk of hemodynamic perturbations.

Our results are supported by prior investigations focused specifically on hemodynamic modulation. For instance, Mounisha et al. (2024) demonstrated a significant attenuation of HR and BP surges during neurosurgical intubation using intracuff alkalinized lignocaine, where maintaining cerebral perfusion stability is critical [13, 14]. Likewise, Assefa et al. (2022) observed reduced heart rate and BP fluctuations during extubation in pediatric patients, attributing the effect to local anesthetic action on airway reflexes [11]. These findings strengthen the case for the use of intracuff lignocaine in both induction and emergence phases for blunting sympathetic overactivity.

In contrast, several studies in the literature have primarily emphasized the post-intubation airway comfort benefits of intracuff alkalinized lignocaine. Sony et al. (2023) conducted a randomized trial demonstrating reduced incidence of sore throat and emergence agitation when lignocaine was used as the cuff inflation medium [12]. Similarly, Navarro et al. (2012) reported significant reductions in coughing, sore throat, and hoarseness in smokers when 2% alkalinized lignocaine was used intracuff, supporting its role in improving patient comfort post-extubation [13]. Furthermore, Gupta et al. (2022) and Barbosa Junior et al. (2024) found alkalinized lignocaine superior to other inflation media (saline, propofol) in minimizing postoperative laryngotracheal morbidity [15,16].

The underlying pharmacologic mechanism is well-documented. Alkalinization of lignocaine increases the proportion of its non-ionized form, enhancing its diffusion across the cuff’s semi-permeable membrane. This leads to effective topical anesthesia of the tracheal mucosa, thereby suppressing afferent nerve stimulation and reducing central sympathetic outflow. This mechanism not only helps blunt acute hemodynamic responses but also minimizes airway irritation during extubation, offering a dual clinical benefit.

The simplicity and non-invasiveness of this method make it a practical and attractive option in routine anesthetic practice. While previous studies have suggested that intracuff alkalinized lignocaine may be cost-effective by reducing postoperative airway complications and minimizing the need for additional interventions or medications, the present study did not conduct a formal cost-benefit analysis to validate this. For instance, Navarro et al. demonstrated a reduction in emergence-related airway symptoms such as coughing and hoarseness, which could translate into decreased postoperative discomfort and resource utilization [13]. Similarly, Sony et al. emphasized that intracuff lignocaine not only improves emergence quality but also reduces recovery time and associated costs in day-care surgical settings [12]. However, these findings should be interpreted cautiously, and future studies should include formal pharmacoeconomic evaluations. Additionally, our study’s observation period was restricted to the first 10 minutes following intubation, capturing only the immediate hemodynamic responses and not postoperative outcomes such as coughing, sore throat, or laryngospasm.

Limitations

Despite the methodological strengths of this randomized, prospective study, such as standardized anesthetic protocols, clearly defined outcomes, and objective hemodynamic measurements, several limitations must be acknowledged. First, the study involved a relatively small sample size (n = 60), which may limit the statistical power and generalizability of the findings. The study population was restricted to ASA I-II patients undergoing elective surgery, and thus, the results may not directly apply to higher-risk populations or emergency settings.

Second, the observation period was limited to the first 10 minutes following endotracheal intubation, capturing only the immediate hemodynamic responses to airway manipulation. As a result, the study does not address longer-term outcomes, such as intraoperative hemodynamic fluctuations or postoperative airway morbidity (e.g., sore throat, cough, or hoarseness), which are also clinically relevant when evaluating intracuff techniques.

Third, while measures were taken to standardize procedures, the study did not incorporate blinding of the anesthesiologist performing the intubation, which could introduce observer bias in the measurement of hemodynamic parameters. Additionally, although the dose of lignocaine used was within safe mucosal limits, the study did not assess systemic absorption or plasma lignocaine levels, nor did it explore the dose-response relationship or alternative concentrations.

Lastly, this investigation did not qualify for mandatory clinical trial registration due to its observational nature, but future interventional studies should consider formal registration to enhance transparency.

These limitations highlight the need for larger, multicenter, and blinded studies with extended follow-up and inclusion of postoperative outcomes to validate and expand upon the preliminary evidence presented here.

Material safety considerations

Although theoretical concerns exist regarding possible chemical interactions between alkalinized lignocaine and the PVC material of endotracheal tube cuffs, current evidence indicates that intracuff lignocaine is generally safe for short-term intraoperative use with standard high-volume, low-pressure PVC tubes. Available data have not demonstrated any significant cuff degradation, dislodgement, or leakage when lignocaine is used for procedures lasting up to two to three hours. The drug is known to diffuse effectively across the cuff membrane without causing structural alterations or compromising its pressure-retaining capacity [13-16].

Furthermore, there is no scientific indication that intracuff lignocaine promotes biofilm formation or microbial colonization within the cuff or lumen. In the present study, no complications such as cuff leak, dislodgement, or allergic reactions were observed during the 10-minute post-intubation observation period. Nonetheless, the potential for long-term effects with prolonged exposure or repeated use remains uncertain. Future investigations assessing the biochemical compatibility, infection risk, and mechanical performance of various cuff materials in the presence of lignocaine would be valuable to further validate its safety profile.

## Conclusions

This prospective, randomized study suggests that inflating the ETT cuff with alkalinized lignocaine attenuates the acute hemodynamic responses associated with laryngoscopy and intubation more effectively than air inflation. Patients in the lignocaine group demonstrated lower and more stable HR, SBP, DBP, and MAP values during the immediate post-intubation period. The technique is simple, noninvasive, and feasible for routine clinical use, particularly in patients undergoing general anesthesia who are at risk of cardiovascular instability. While the findings are promising and consistent with prior literature, the study’s limited sample size and short observation window warrant cautious interpretation. Larger, multicenter trials with extended follow-up and postoperative airway assessments are recommended to confirm the safety, reproducibility, and broader applicability of intracuff alkalinized lignocaine in contemporary anesthetic practice.
